# Endoscopic Ultrasonography In Diagnostic Endohepatology: The Hepatologist’s Swiss Army Knife

**DOI:** 10.5152/eurasianjmed.2023.23372

**Published:** 2023-12-01

**Authors:** Fatih Albayrak

**Affiliations:** Department of Gastroenterology, Atatürk University Faculty of Medicine, Erzurum, Turkey

**Keywords:** EUS, chronic liver disease, Doppler EUS, contrast enhanced EUS, EUS-SWE (EUS-elastography), EUS-guided liver biopsy (LB), EUS-guided sampling for focal liver lesions

## Abstract

In recent years, significant advances have been made in endoscopic ultrasonography (EUS) processors, transducers, and the instruments used. In parallel with these developments, the importance of EUS in the diagnosis, treatment, and management of chronic liver diseases has increased considerably. One of the most important advantages of EUS is that the ultrasound probe can examine the liver area under examination from a very close distance. Additionally, EUS provides the ability to provide excellent spatial files and real-time images; Doppler, elastography, and contrast methods are also widely used to increase diagnostic accuracy in EUS evaluations. Endoscopic ultrasound-guided procedures are an alternative method used when percutaneous approaches are not feasible. Endoscopic ultrasound-based advanced applications such as EUS-guided liver biopsy (LB), EUS-guided ascites assessment, the use of EUS in the evaluation of focal lesions of the liver, and EUS portal pressure gradient (EUS-PPG) measurements are becoming increasingly common and important. As a result of the developments in EUS devices and instruments, it is possible to perform all diagnostic procedures in hepatology with EUS. In other words, EUS can provide all the imaging and interventional procedures that a hepatologist would require for diagnosis in a single session, without the need for any other expertise other than the gastroenterologist (such as a radiologist, interventional radiologist, or surgeon). This is called the single-procedure diagnostic approach, or “all in one” in hepatology. From a hepatologist’s perspective, EUS can be thought of as the “hepatologist’s Swiss army knife.”

## Introduction

Conventional tools used in the evaluation of chronic liver disease include ultrasonography (USG), which is a harmless and low-cost method; computed tomography (CT) imaging, which cannot be used in some cases due to high radiation exposure; and magnetic resonance imaging (MRI). The diagnosis and treatment of chronic liver disease and its main complications continue to evolve with the advancement of medicine and technology. With the new techniques and possibilities developed in the field of endoscopy becoming available in hepatologic approaches, the concept we call “endohepatology or endo-hepatology” began to resonate in the medical world.^1,2^ In this article, we elucidate the usage areas of diagnostic endoscopic ultrasound (EUS) in endo-hepatology.

The development and availability of EUS have made it an interesting and important method in the field of hepatology, especially in end-stage liver diseases, as in other fields, at every stage from diagnosis to treatment. The most important advantages of EUS are that it can combine both endoscopy and sonography features in a single hybrid device and that more detailed and accurate images can be obtained by bringing the probe closer to the liver, which is the main area of interest. Additionally, EUS provides the ability to provide excellent spatial files and real-time images. Supplementary techniques like contrast-enhanced (CE) EUS, duplex-color Doppler, and elastography increase the importance of EUS day by day. When percutaneous approaches to the liver cannot be performed for various reasons, approaching the liver using the gastrointestinal tract with EUS emerges as a realistic and feasible solution to these problems. Endoscopic ultrasound-based advanced applications such as EUS-guided liver biopsy (LB), EUS-guided ascites assessment, the use of EUS in the evaluation of focal lesions of the liver, and EUS portal pressure gradient (EUS-PPG) measurements are the main examples of these approaches. As a result of the developments in EUS devices and instruments, it is possible to perform all diagnostic procedures in hepatology with EUS. This is called the single-procedure diagnostic approach, or “all in one” in hepatology. From a hepatologist’s perspective, EUS can be thought of as the “hepatologist’s Swiss army knife.”

## Clinical and Research Consequences

### Interventional Diagnostic Procedures

#### Endoscopic Ultrasound-Guided Liver Biopsy

Although the recent development of noninvasive methods for the evaluation of liver fibrosis has called into question the necessity of LB, its position as the gold standard for the evaluation of hepatic fibrosis has not yet changed. Conventional liver biopsy is divided into 2 types: percutaneous liver biopsy (PC-LB), which is performed directly through the skin with a needle, and transjugular liver biopsy (TJ-LB), which uses a cannula advanced through the jugular vein for biopsy collection. Both are techniques for collecting liver tissue for histopathologic examination in the diagnosis and treatment of parenchymal liver diseases. When a percutaneous approach is used, the localization of the liver biopsy is determined by ultrasonographic imaging, and a 16- or 18-gauge needle is used to take the biopsy. The presence of conditions such as obesity, ascites, and coagulopathy can make percutaneous biopsy difficult to perform. While PC-LB or TJ-LB is currently considered the standard approach for liver biopsy, EUS-guided liver biopsy (EUSLB) is a new approach that stands out with its unique advantages. It has been reported that EUS visualization during fine needle aspiration (FNA) application provides high sensitivity for the detection of focal lesions in the liver and biopsy application.^1,3^ Adequate tissue size is essential for accurate diagnosis, reliable grading, and successful staging of liver diseases based on biopsy specimens. For this reason, the American Association for the Study of Liver Diseases recommends that for a liver biopsy sample to be considered adequate, the sample must contain 11 or more complete portal tracts of at least 2-3 cm in length.^4^ Although EUS-LB is an emerging method, it shows similar success rates with conventional biopsy methods in terms of both histologic performance and complete portal tract acquisition.^5^ Endoscopic ultrasound-liver biopsy, which has been found to be safe in many studies, can be performed by most endoscopists if the patient’s platelet count is above 50 000, the INR value does not exceed the 1.5 threshold, and there are no major ascites.^5,6^ Compared to conventional methods, EUS-LB provides accurate localization and identification of the lesion due to the imaging it provides. It also allows evaluation of both liver lobes in a less invasive manner. From a patient welfare perspective, EUS-LB is more easily tolerated, has a faster recovery, and has fewer complications, making it a preferable option.^5-7^ Endoscopic ultrasound is a versatile tool that allows many different procedures to be performed at once. It not only allows the target tissue to be visualized continuously under Doppler ultrasonography but also allows samples to be taken from both lobes. In addition to these, portal pressure measurement, gastroesophageal varices scanning, and many other endoscopic functions can be applied under the EUS concept.^8^ In addition to all these advantages, there are also disadvantages such as the need for deep sedation, a high financial burden that reduces accessibility, and the scarcity of centers with the expertise needed to be able to carry out this procedure successfully, although this is increasing day by day. Endoscopic ultrasound-LB can be used for varicose vein screening, EUS-PPG measurement, focal liver biopsy, and evaluation of surrounding tissues, including lymph nodes. However, its use in patients with ascites and obesity is very limited.^9^ Endoscopic ultrasound-LB is a safe and effective procedure that achieves diagnostic specimen sampling proficiency comparable to PC-LB and TJ-LB. Further prospective studies are needed to determine the cost-effectiveness of each modality and to characterize the patient population in which EUS-LB can be used more efficiently.^8^

#### Portal Pressure Measurement Under Endoscopic Ultrasound Guidance

Portal hypertension (PHT), which is both a cause and consequence of cirrhosis and noncirrhotic portal hypertension, is a condition with a high degree of morbidity and mortality that occurs as a result of an increase in the pressure of the portal venous system that carries blood to the liver for various reasons. Quantitatively, PHT can be detected by a hepatic venous pressure gradient (HPVG) > 5 mm Hg.^10^ Portal hypertension is one of the most important complications of cirrhosis, and the level of PHT is closely correlated with the clinical status of cirrhosis. Therefore, portal pressure measurements not only reflect the level of PHT but also guide the clinician in determining the level of cirrhosis and predicting the prognosis as the best indicator of it. Although HVPG is the gold standard method for measuring portal pressure, the difficulties in its practical application limit its use.^2^ Other disadvantages of HVPG include its inability to evaluate presinusoidal and prehepatic PHT and its indirect, rather than direct, representation of portal vein (PV) pressures.^8,11^ Since the gastrointestinal tract, the site of application of EUS, is a close neighbor of the portal circulation, the use of EUS in PV catheterization allows efficient observation of the area of interest and greatly facilitates PV application. The portal pressure gradient (EUS-PPG) is calculated by determining the hepatic venous pressure under EUS guidance and determining the difference between it and the portal pressure value. The aforementioned hepatic venous pressure and portal pressure values are measured by entering these vessels by transgastric puncture via EUS-PPG. The manometry device to be used for this measurement should include a noncompressible tube, heparinized saline, a digital manometer, and a 22-25G needle for FNA.^5,12^ Endoscopic ultrasound-PPG has obvious advantages over conventional HPVG, such as being less invasive, reducing the patient’s radiation exposure, and showing PHT more accurately by directly calculating PPG.^11^ Based on the literature, EUS-PPG has a very high success rate, ranging from 96% to 100%. Despite this very high success rate, it cannot be used in patients with the most advanced stages of portal hypertension with platelet counts above 50 000/L, extended INR time, and the presence of a large volume of ascites in the abdomen.^5^ In addition, the effects of other parameters (e.g., endoscope pressure, scope position, and tension on the scope) on the reliability and repeatability of pressure measurements are other limitations.

#### Endoscopic Ultrasound-Guided Portal Venous Blood Sampling

For interventions through the stomach or duodenum, the echoendoscope can be positioned very close to the PV. Intrahepatic PV via the hepatic parenchyma is the most commonly targeted site; less frequently, extrahepatic PV via the duodenum is targeted. Transgastric and transhepatic approaches are more advantageous than trans-duodenal approaches in terms of safety.^9,13^ The application of EUS for transgastric puncture of the portal vein has provided a minimally invasive alternative for practitioners previously confined to surgical or Transhepatic Intravenous Portosystemic Shunting options.^2^ Poor prognosis is suspected in the presence of malignant portal vein thrombosis, and therefore FNA confirmation is required to differentiate between benign and malignant thrombus. One of the most important considerations during the evaluation of portal vein thrombosis in cirrhotic patients is to determine whether the thrombosis is of malignant origin when hepatocellular carcinoma (HCC) is suspected. Although endoscopic ultrasound-guided fine needle aspiration (EUS-FNA) is among the options that clinicians may consider in such cases, it is not one of the most frequently reported methods.^14,15^ Keeping the probe close to the liver hilum increases the value of this procedure by enabling a safer, more accurate cytologic diagnosis.^14^

### Noninterventional Diagnostıc Procedures

#### Evaluation of the Hepatic Parenchyma

**Figure 1.**
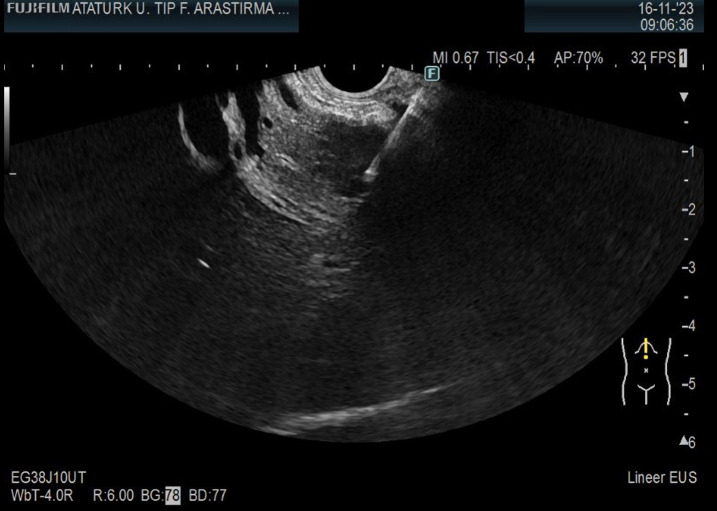
Endoscopic ultrasound-guided liver lesion biopsy performed in a patient where percutaneous biopsy could not be performed at the portal hilus.

Transabdominal USG, CT scan, or MRI are the clinician’s first-line modality for the evaluation of liver parenchyma or focal lesions. Endoscopic ultrasound has important additional features that can contribute to diagnosis and treatment, and its use in these indications is increasing.^9,16,17^ The use of EUS in liver diseases has many differences compared to conventional USG/CT/MRI methods and has significant advantages, especially in the evaluation of hepatic parenchyma. When evaluating the liver parenchyma with transabdominal ultrasound, the tissues between the skin and the liver parenchyma interfere with obtaining a reliable image. In the EUS method, problems that prevent imaging, such as intestinal rings and ribs, are eliminated, and a more detailed and accurate image is obtained due to the fact that the probe can be held much closer to the parenchyma. In addition to this high-quality image, EUS provides better evaluation of deep structures through its probe.^17^ In accordance with the EUS scope’s location, different structures can be distinguished, including: The liver hilum, the ligamentum venosum, the caudate lobe (segment I), the inferior vena cava, the right lobe (segments V and VIII), the left lateral segments (segments II and III), the left PV, and the umbilical part of the ligamentum teres can all be assessed in EUS applications conducted from the stomach. On the other side, segments VI and VII, hepatoduodenal ligament structures, PV and hepatic artery branches, liver hilum, and segmental portions of the right PV and hepatic artery can all be seen with the duodenal bulb in EUS applications.^17-19^ Endoscopic ultrasound has superior performance compared to other conventional imaging modalities (US, CT, and MRI) when evaluating focal lesions of the liver smaller than 1 cm. This increases the importance of EUS, especially in metastatic cases where the primary source is not the liver.^19,20^ The use of EUS to differentiate benign from malignant metastatic lesions of the liver leads the clinician to the correct diagnosis in as high as 82% of cases. By evaluating parameters such as lesion shape, echogenicity, homogeneity or heterogeneity, and size, it is possible to differentiate malignant from benign. The lesion must meet at least 3 criteria to be considered neoplastic: (1) absence of an isoechoic/mildly hyperechoic center; (2) post-acoustic enhancement; (3) presence of disruption of adjacent structures; (4) hypoechogenicity (mild or marked); and (5) size greater than 10 mm.^9,21^ A study by Singh et al,^19^ including data from 132 patients, showed that EUS has a far superior diagnostic accuracy rate of 98% compared to the 92% accuracy rate of CT in detecting metastatic disease (*P* = .0578). Endoscopic ultrasound clearly demonstrates the need for it in clinical practice, especially by demonstrating its success in showing small lesions (less than 3 mm) that even detailed examinations such as CT and MRI cannot detect.^22^ In a study by Okasha et al,^23^ EUS failed to detect only 7 lesions that MRI and CT detected (6 of these lesions were metastases), while CT and MRI methods failed to detect 58 lesions (42 of which were metastases) that EUS was able to detect.^23^ In addition to its advantage in the detection of these lesions, EUS, compared to MRI and CT, has demonstrated its diagnostic power by providing not only images but also the possibility of FNA biopsy (Figure 1). 

Endoscopic ultrasound is an evaluation method that has made significant contributions on its own, but its diagnostic success has been further enhanced by various developments. Especially contrast-enhanced EUS (CE-EUS) methods, which take advantage of the different blood supply structure of malignant lesions compared to benign lesions, are of great importance in this sense. Contrast-enhanced-EUS methods consist of 2 types: CE-EUS with Doppler (CE-EUS-D), in which Doppler imaging is used in addition to the use of contrast, and CE-EUS with harmonic imaging (CE-EUS-H), in which contrast is combined with harmonic imaging. The contrast agents used for these methods consist of microbubbles that work by trapping perfluorobutane or sulfur hexafluoride substances in a lipid shell.^2,24^ The principles of using these microbubbles are based on the liver’s blood supply system, which consists of 3 phases: arterial phase, portal venous phase, and late phase until the contrast agents are cleared.^9,25^ In a study by Oh D et al^26^, it was shown that only 73.3% of patients with metastases could be detected with conventional EUS, while a very high success rate of 93.3% was achieved when contrast-enhanced EUS methods were used. Considering all of the above, CE-EUS is a more successful option for the detection of liver metastases than conventional EUS and CT scanning.^27^ The advantages of CE-EUS over CT and MRI are summarized as follows: (1) imaging is real-time and continuous; (2) unlike the contrast-enhanced options of these modalities, contrast is not excreted from the kidneys, so it can be used in patients with renal insufficiency; (3) contrast is limited to the vascular area, so imaging of the vascular system is prolonged; (4) imaging is more localized, resulting in higher resolution; (5) improves the feasibility of biopsy; and (6) is more successful in detecting lesions smaller than 1 cm.^9^

#### Endoscopic Ultrasound Doppler Assessment

**Figure 2.**
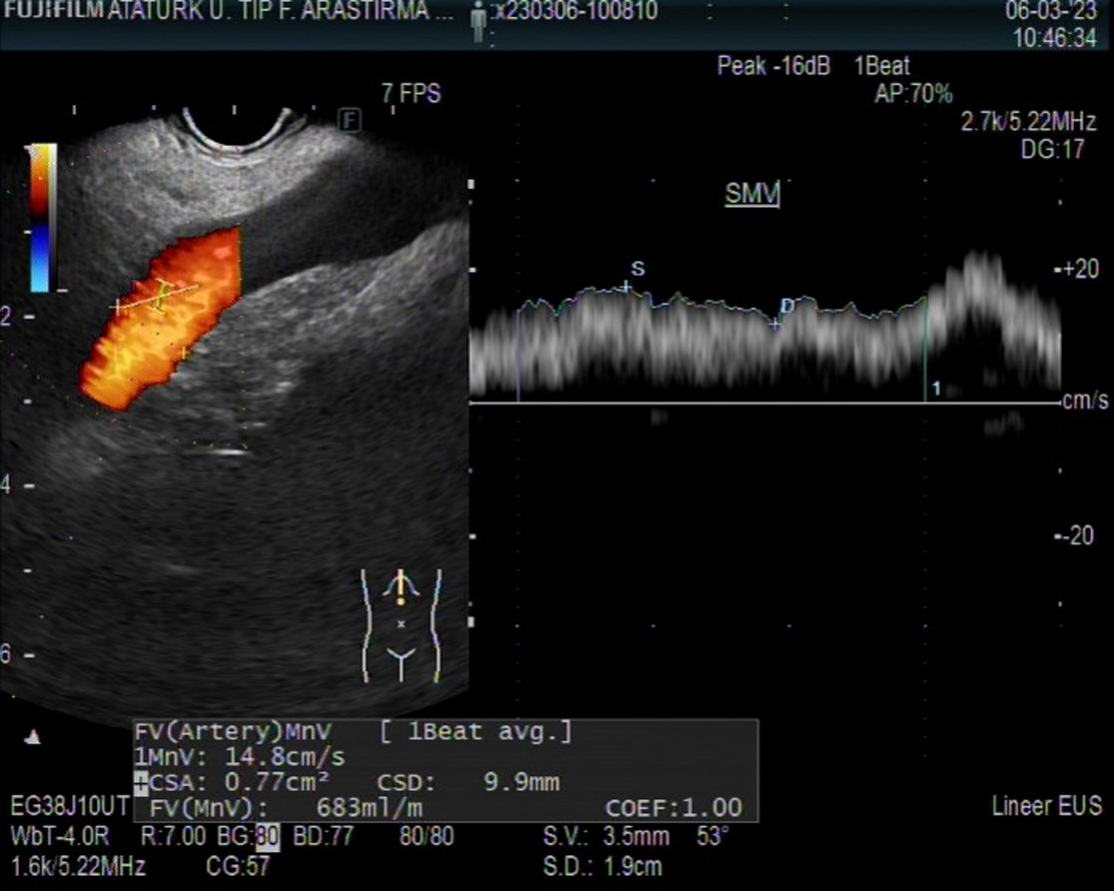
Superior mesenteric vein Doppler spectrum and flow velocity.

Doppler techniques are very useful in chronic liver diseases. Among them, the color Doppler method enhances the capacity of endosonography to detect blood vessels around the gastrointestinal tract, while duplex Doppler is used to examine the portal, hepatic, superior mesenteric, and splenic veins and the hepatic artery by measuring maximum or average velocities (Figure 2). Color Doppler significantly facilitates the differentiation of blood vessels from nonvascular structures, especially in the gastrointestinal wall and adjacent structures (e.g., lymph nodes). Duplex Doppler endosonography is a method that can also be used to measure blood flow parameters in hemodynamic studies.^28-30^ Resistance index and pulsatile index are Doppler indices commonly used to assess arterial vascular resistance in the vascular bed and compare systolic and diastolic flow. In the normal hepatic arterial system, the high resistance caused by chronic liver disease is not present, so there is a low resistance flow.^30^ Hepatic vascular flow abnormalities are the signs that alert the clinician in the early stages of liver diseases. For example, chronic liver disease has been found to be associated with decreased hepatic artery resistance indexes in Doppler US measurements of patients.^30,31^ Chronic liver disease has also been reported to alter the Doppler waveform pattern of the portal vein, indicating vascular compliance in the livers of patients.^30^ Hemodynamic assessments using the Doppler US are becoming increasingly important in the evaluation of chronic liver diseases.^3^

**Figure 3.**
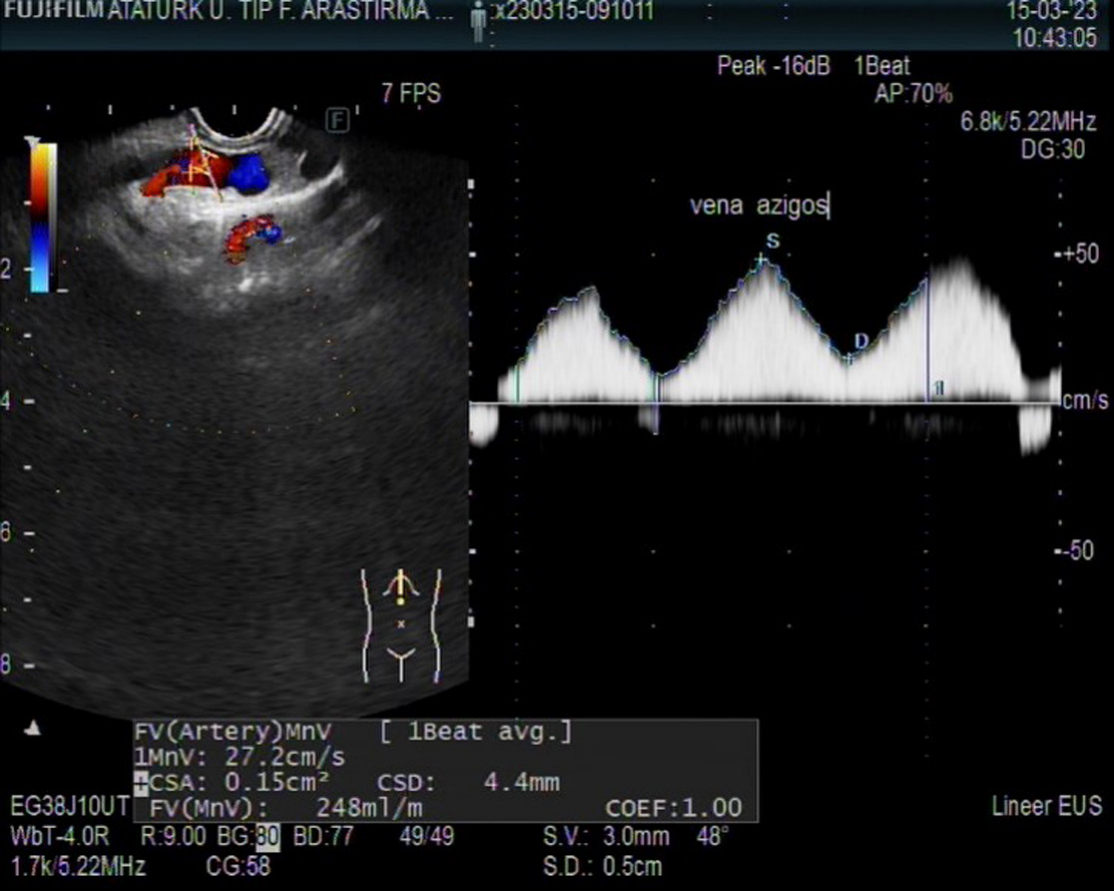
Vena azygos velocity and Doppler spectrum.

Endoscopic ultrasound is also a sensitive and specific method when used to diagnose portal venous system thrombosis.^32^ The flow rate and characteristics of the azygos vein are evaluated with Doppler EUS (Figure 3). Changes in azygos vein flow before and after treatment for esophageal varicose veins can be evaluated with EUS Doppler to determine the risk of rebleeding and the patient’s treatment.^33^ Doppler with EUS is superior to upper gastrointestinal endoscopy in terms of sensitivity in detecting esophageal varices (EVs) and gastric varices, and it can also be used to assess the possibility of rebleeding. The following items will help to demonstrate the superiority of EUS: (1) EUS allows observation of collaterals and feeder vessels in the region of interest, which is important for assessing rebleeding; (2) hematocystic staining on EVs, which are at high risk for variceal rupture, can be visualized on EUS as saccular aneurysms; (3) one of the advantages of EUS, digital image analysis, can be used to determine the cross-sectional area of EVs in the distal esophagus. If this area is 0.45 cm^2^ or more, this has a sensitivity of 83% to indicate the risk of future rebleeding; (4) determining the para-esophageal diameter after esophageal variceal band ligation, which is one of the treatment modalities for EVs and can be detected by EUS, is very successful in predicting the risk of recurrence (the cutoff of 4 mm has a sensitivity of 70.6%);^9,34^ and (5) Endoscopic patent inflowing perforating veins can be detected with color Doppler EUS, and detection of these veins has a predictive value for early variceal recurrence.^35^

The left gastric vein that EUS can observe is closely related to the size of gastric varices, so EUS allows us to evaluate gastric varices by visualizing the left gastric veins.^36^ The blood flow velocity of the veins can also be determined by means of a color Doppler EUS examination. If the hepatofugal flow velocity of the left gastric vein is found to be above 12 cm/s with this method, this indicates a risk of early recurrence of previously treated esophageal varices.^37^

#### Endoscopic Ultrasound Tıssue Stıffness Assessment

With the development of imaging techniques in medicine, elastography methods, including vibration-controlled transient elastography (VCTE), have gained an important place among the options for the evaluation of fibrosis in the liver. Vibration-controlled transient elastography is a method based on measuring the speed of waves produced by mechanical action. The method of diagnosing disease by generating waves through mechanical action is very old. The best known of this method is “Sensation des flots.” Vibration-controlled transient elastography has become indispensable in clinical use with its ability to assess liver stiffness, one of the most important findings of liver fibrosis. With this essence, VCTE is the most widely used point of care to assess liver fibrosis among noninvasive techniques.^38,39^ Despite all these good features, the unsuccessful use of VCTE in individuals with narrow intercostal spaces and morbid obesity is an obstacle that has not yet been overcome.^38,40^

Elastography point quantification (ElastPQ) enables noninvasive assessment of liver fibrosis using ultrasound-based shear wave elastography (SWE).^38,41^ This technique combines elastography, which is of great importance in the evaluation of liver fibrosis, with a conventional ultrasound device. With the power of this combination, SWE offers significant advantages in several clinical circumstances, such as measuring the stiffness of the areas to be examined and performing B-mode examination simultaneously. Shear wave elastography is used through a quantitative evaluation method. Values measured as shear wave velocity (Vs) are then displayed in meters per second (m/s). The stiffness of a lesion is either determined as Vs in m/s across the tissue or as the strain modulus in kilopascals (kPa). The strain modulus (or Young’s modulus) is calculated by the following equation (assuming a tissue density of 1 g/cm^3^ and a Poisson’s ratio of 0.5):^42^

(kPa) = 3 Vs^2^

**Figure 4.**
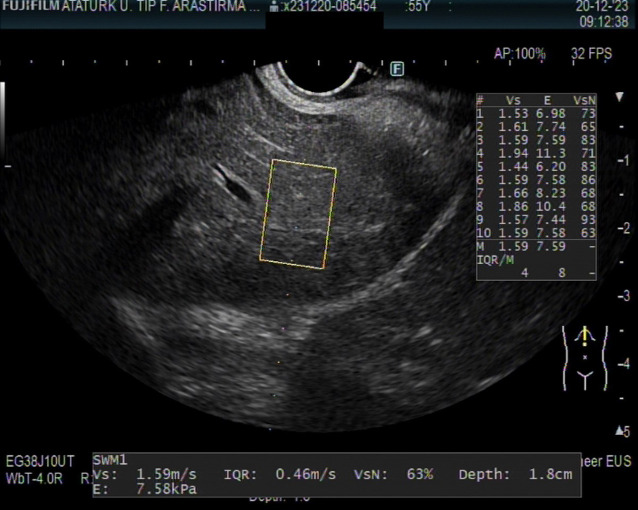
Evaluation of liver stiffness with endoscopic ultrasound shearwave elastography.

Endoscopic ultrasound elastography, which is an advanced use of elastography in this field and is still evolving, is now available as an option on almost all new echoendoscopes. Unlike the traditional transabdominal procedure, endoscopic elastography (its variant endoscopic ultrasound shear wave elastography (EUS-SWE)) provides higher accuracy and better quality images of liver parenchyma and fibrosis by targeting the probe through the stomach, which is located next to the liver, rather than outside the body (Figure 4). In cases where transabdominal elastography is hampered by central adiposity, narrow rib spaces, or large amounts of ascites, the fact that EUS-SWE is not affected by these limitations makes it even more important.^43^ A study by Kohli DR et al^44^ published in 2023, evaluated the diagnostic success of EUS-SWE and VCTE on liver biopsies of 42 patients and showed that EUS-SWE is a safe and reliable method for evaluating liver fibrosis. Although the similar cross-validated AUROCs of VCTE and EUS-SWE when evaluating cirrhosis and fibrosis suggest that there is little difference between them, VCTE could not be successfully completed in 8 of 42 patients in the study, but liver stiffness was successfully measured by EUS-SWE in these patients. However, we need more data so that the BAVENO VII’s “rule of 5” based on VCTE can be transferred to EUS-SWE. If data increases to show that EUS-SWE results are similar to or better than VCTE, EUS-SWE will become an indispensable tool in chronic liver diseases.^2^

**Figure 5.**
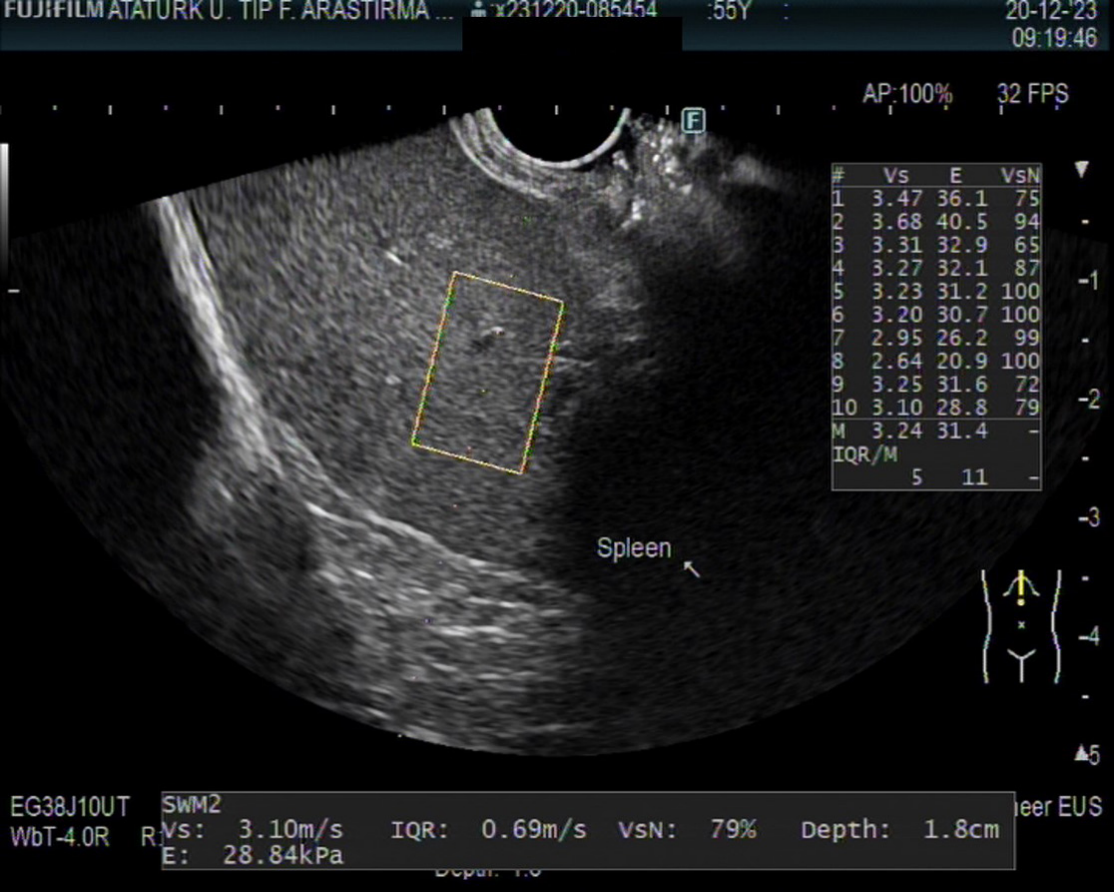
Evaluation of spleen stiffness with endoscopic ultrasound shearwave elastography.

Theoretically, not only the stiffness of the liver parenchyma but also the stiffness of the spleen can be easily measured with EUS-SWE. Although spleen hardness can be measured with VCTE, this requires special equipment and increases costs. In addition, it is very difficult to measure with VCTE, especially in patients without splenomegaly. However, spleen stiffness can also be easily measured by EUS-SWE (Figure 5). It is clear that more data on these issues is needed before EUS-SWE can be incorporated into clinical practice.

#### Endoscopic Ultrasound Biliary Assessment

Biliary pathologies are an important area of interest in endohepatology, and the biliary tract (CBD) is the most essential anatomical structure used in the evaluation of biliary pathologies. The size of the CBD is also very important in these pathologies, and this parameter can be modified by many different factors, such as choledochal cysts, age of the patient, any previous surgical manipulation of the site, use of narcotic drugs, obstructions, and many more. There is an ongoing debate as to what the upper limit of CBD diameter should be, but commonly, a diameter of 7 mm is considered normal in patients in general, while values above 10 mm in patients who have undergone a cholecystectomy procedure indicate the presence of pathology.^45,46^ Although transabdominal ultrasonography is generally used as the first diagnostic technique for detecting biliary pathologies, it generally does not provide sufficient information due to its low sensitivity. As a result of this low sensitivity, the exact cause cannot be determined in one-third of patients. In addition, ampulla tumors are often not evaluated in transabdominal ultrasonography examinations because of the intestinal gas present in them.^47^ In evaluating a dilated bile duct, CT scanning is very sensitive, especially in detecting pancreatic tumors larger than 2 cm. However, in tumors smaller than 2 cm, sensitivity may decrease to 77%.^47,48^ Magnetic resonance cholangiography (MRCP) is considered the gold standard among noninvasive methods for its success in evaluating the biliary tree. The MRCP is highly sensitive in CBD stones. However, its sensitivity drops below 50% in stones smaller than 3 mm.^47^

Endoscopic ultrasound examination has been used in pancreaticobiliary evaluations in recent years and has become a method of increasing importance as it has been found to be highly accurate in detecting stones in extrahepatic ducts.^49^ Meta-analyses of the ability of EUS to detect CBD stones have shown that EUS has a strong sensitivity of 94% and a high specificity of 95%.^49,50^ One of these meta-analyses included 3532 patients and showed that EUS was able to detect malignant biliary strictures with a sensitivity of 78% and a specificity of 84%.^50^ In ampullary lesions, deeper invasion of the lesions can be evaluated by EUS examination, and staging of ampullary tumors can also be performed.^47^ The use of EUS examination in the management of pancreatobiliary diseases is very useful for systematic examination of the extrahepatic parts of the biliary tree, duodenal wall, and periampullary system. Although EUS is an invasive test, complication rates are low, and it is well tolerated.^47,51^

The biggest advantage of MRCP compared to EUS is that it is a noninvasive method and can be used in patient groups that cannot tolerate EUS.^52^ However, examining MRCP images is a complicated method requiring technical expertise and a high degree of patient cooperation, and patients should not be claustrophobic.^52,53^ As for the disadvantages of EUS, the possible air bubbles or stents in the bile duct can lead to false negative results.^52^ The biggest challenge with EUS imaging is interobserver differences in assessment. Endoscopic ultrasound allows the user to achieve a very high resolution by allowing the endoscope probe to be very close to the internal tissues of interest. This high resolution makes EUS superior to MRCP in detecting small stones. In addition, if a stone or pathology requiring ERCP is detected during EUS, ERCP can often be performed without the need for additional sedation.^52^

### Limitations of Endoscopic Ultrasound

When used for diagnostic purposes, the minimally invasive aspect of EUS can be considered a disadvantage. The experience and meticulousness of the endosonographer performing liver evaluations are critical for diagnosis and treatment. Fatty infiltration and calcification in the area to be imaged, the presence of fibrosis, and air bubbles in the biliary tract also prevent obtaining healthy images with EUS. Changing anatomy (e.g., gastrectomy, presence of pharyngeal diverticula, or tight stenosis) may also limit EUS performance. Furthermore, diagnostic accuracy is reduced when the lesion is located in the right lobe of the liver or below the diaphragmatic dome. The major limitation of the existing literature on EUS in liver diseases is that most of the studies do not include enough patients, are single center, and are generally nonprospective and nonrandomized.^17^

## Conclusion

Endoscopic ultrasound has become very important in the diagnosis of liver diseases by enabling the clinician to evaluate liver parenchyma and liver lesions with real-time, uninterrupted, high-resolution visualization through gastric or duodenal examinations. Doppler, elastography, and contrast methods used in transabdominal ultrasound are also widely used to increase diagnostic accuracy in EUS evaluations. Additionally, EUS reduces the complication rates and increases the success rates of methods such as liver biopsy and portal pressure measurement by guiding the clinician with real-time imaging. In other words, EUS can provide all the imaging and interventional procedures that a hepatologist would require for diagnosis in a single session, without the need for any other expertise other than the gastroenterologist (such as a radiologist, interventional radiologist, or surgeon). This is called the single-procedure diagnostic approach, or “all in one” in hepatology. We can use the phrase “hepatologist’s Swiss army knife” for EUS, which does a lot of work in a single session.
